# Supplementary material for: Dust-wind interactions can intensify aerosol pollution over eastern China
Source: Nat Commun. 2017 May 11;8:15333. doi: 10.1038/ncomms15333 (PMC5437281; doi:10.1038/ncomms15333)
Supplement: Supplementary Information — Supplementary Figures [file ncomms15333-s1.pdf]

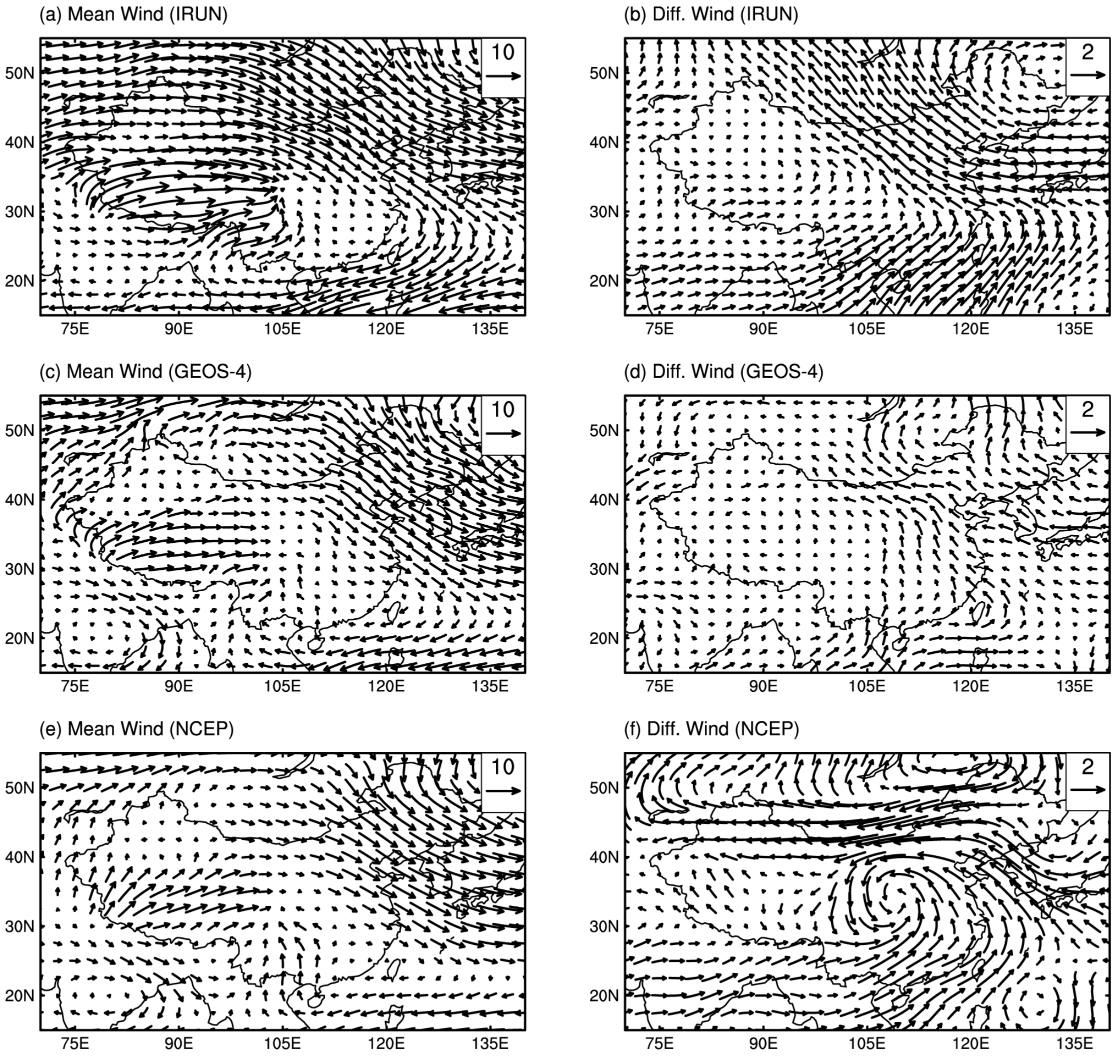

Supplementary Figure 1. Climatological mean and anomalous winds. Climatological mean wind fields (unit: m s^{-1}) in December-January-February (DJF) from (a) the IRUN simulation, (c) GEOS-4 assimilated meteorological fields for 1986–2006 and (e) NCEP/NCAR (National Centers for Environmental Prediction/National Center for Atmospheric Research) reanalysis data for years of 1980–2016. Composite differences in wind field (unit: m s^{-1}) between weak wind and normal conditions calculated based on 850 hPa wind speed from (b) IRUN, (d) GEOS-4 and (f) NCEP/NCAR meteorological fields.

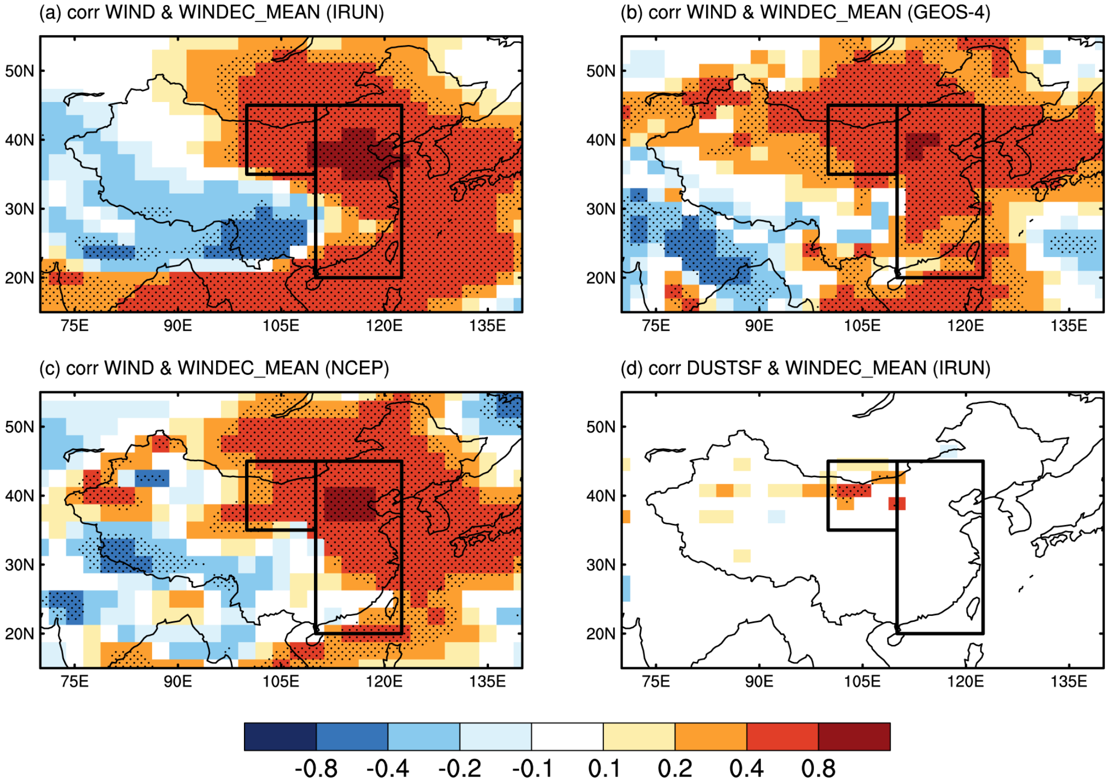

Supplementary Figure 2. Correlation of grid winds and wind speed over eastern China. Correlation coefficients between wind speed at 850 hPa and wind speed averaged over eastern China (110–122.5°E, 20–45°N) in DJF from (a) the 150-yr IRUN simulation, (b) the GEOS-4 assimilated meteorological fields for 1986–2006, and (c) the NCEP/NCAR reanalysis data for years of 1980–2016 (a). (c) Correlation coefficients between dust emissions and wind speeds averaged over eastern China in DJF from the 150-yr IRUN simulation. The region boxed is used to represent the Gobi Desert region (100–110°E, 35–45°N, left box) and eastern China (110–122.5°E, 20–45°N, right box). The dotted areas indicate statistical significance with 95% confidence.

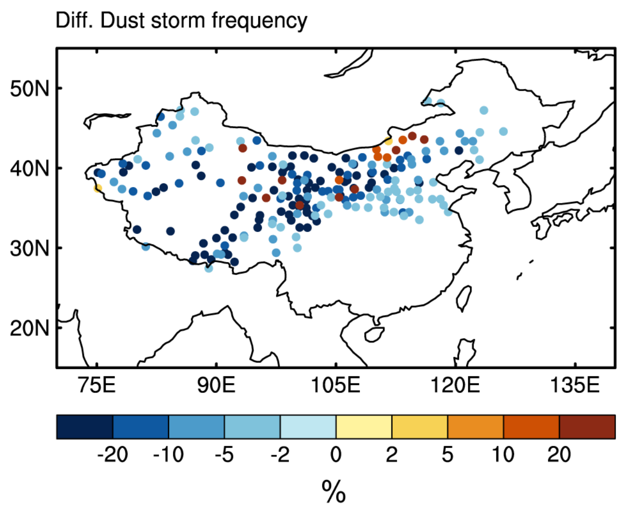

Supplementary Figure 3. Differences in observed dust in weak wind conditions. Composite differences in observed dust storm frequency (unit: %) over 753 sites in China between weak wind and normal conditions based on 850 hPa wind speed from NCEP/NCAR meteorological fields for 1981–2015. Dust storm frequency is defined as dust storm days per examined days. Sites without dust day in DJF are not shown.

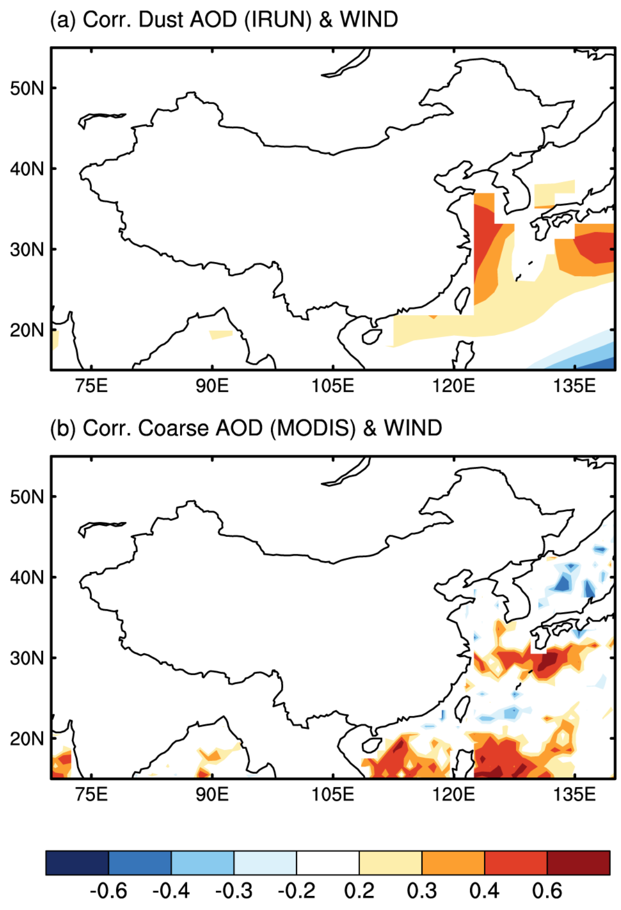

Supplementary Figure 4. Correlation between dust AOD and wind speed. (a) Correlation coefficients between simulated dust aerosol optical depth (AOD) at 550 nm and wind speed at 850 hPa averaged over eastern China (110–122.5°E, 20–45°N) in DJF from the 150-yr IRUN simulation. (b) Correlation coefficients between observed coarse AOD at 550 nm and wind speed at 850 hPa averaged over eastern China in DJF between 2001 and 2016. Observed AOD is from Moderate Resolution Imaging Spectroradiometer (MODIS) aerosol products of the Terra satellite. Observed wind speed is obtained from NCEP/NCAR reanalysis data. Coarse mode AOD is calculated from total AOD (which was available) minus the fine mode fraction, which is only available over the ocean.

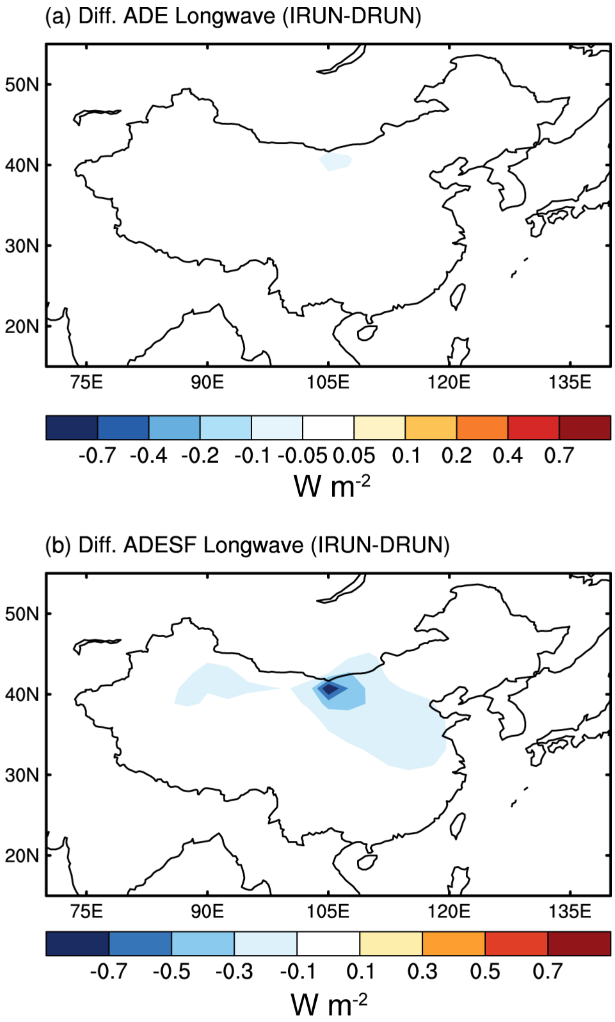

Supplementary Figure 5. Dust induced changes in longwave radiative effect. Changes in longwave aerosol direct radiative effect (unit: W m^{-2}) (e) at the top of the atmosphere and (f) the surface, respectively, between weak wind and normal conditions due to the interannual variations in dust emissions.

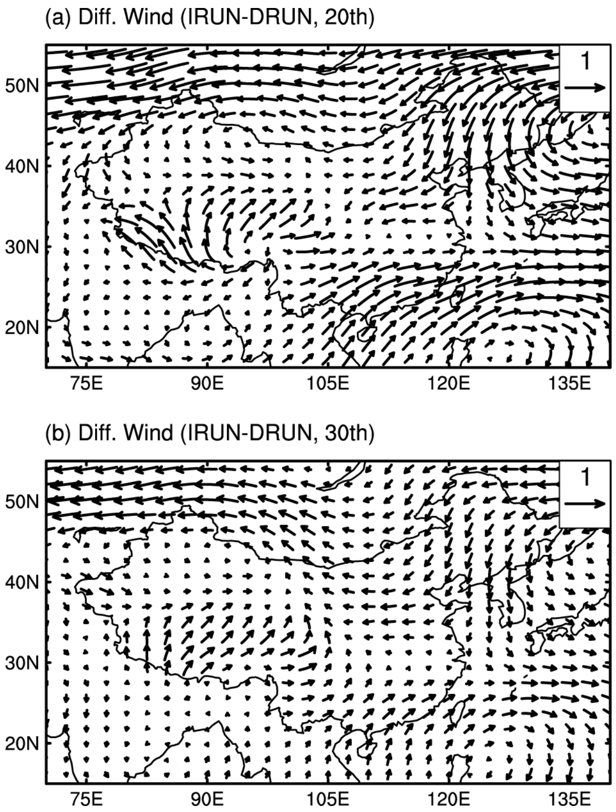

Supplementary Figure 6. Dust induced changes in wind fields based on 20th and 30th thresholds. Changes in wind fields (unit: mg m^{-2}) between (a) 20th and (b) 30th weak wind and normal conditions due to the interannual variations in dust emissions.

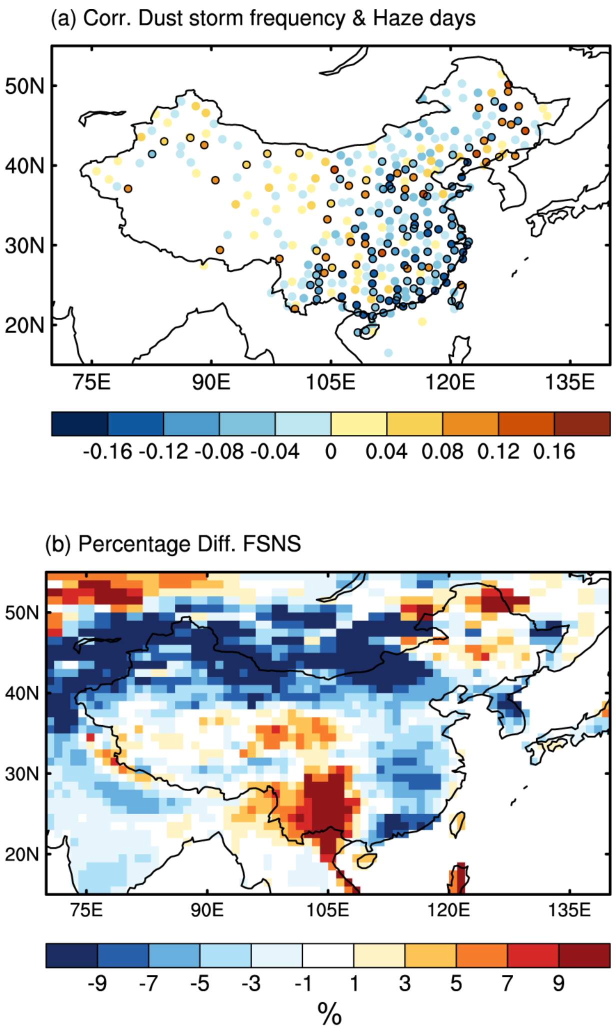

Supplementary Figure 7. Correlation of observed dust and haze and changes in shortwave flux. (a) Correlation coefficients between dust storm frequency averaged over the Gobi Desert and haze days (defined as days with observed atmospheric visibility less than 10 km and relative humidity less than 90%) for 1981–2015. Sites with outline indicate statistical significance with 95% confidence. Observed visibility data are derived from National Climatic Data Center (NCDC) Global Summary of Day (GSOD) database. (b) Relative differences in surface net shortwave flux (FSNS, unit: %) between weak wind and normal conditions based on NCEP/NCAR wind speed. The shortwave flux data are derived from the Clouds and the Earth's Radiant Energy System (CERES) data set for years 2001–2016. Positive values represent net downward fluxes. The relative differences are calculated by $(FSNS_{Weak} - FSNS_{Normal}) / \overline{FSNS_{Normal}}$, which remove spatial variability of the variable.
